# Modulating the lung–gut axis: A randomized controlled trial of Xie Bai Zeng Ye decoction on intestinal flora and mucosal immunity in children with post-infectious cough

**DOI:** 10.1097/MD.0000000000045819

**Published:** 2025-11-28

**Authors:** Jing Luo, Dan Zhang, Guoping Zhan, Yi Ding

**Affiliations:** a Department of Traditional Chinese Medicine, Shenzhen Qianhai Shekou Free Trade Zone Hospital, Shenzhen, Guangdong Province, China; b Department of Pharmacy, Shenzhen Qianhai Shekou Free Trade Zone Hospital, Shenzhen, Guangdong Province, China; c Department of Rehabilitation, Changsha Social Work College, Changsha, Hunan Province, China.

**Keywords:** gut microbiota, post-infectious cough, serum D-lactic acid, short-chain fatty acids, Xie Bai Zeng Ye Decoction

## Abstract

**Background:**

This study aimed to evaluate the clinical efficacy of Xie Bai Zeng Ye Decoction (XBZY) in the treatment of post-infectious cough in children with lung heat and Yin deficiency, and to investigate its effects on gut microbiota composition and intestinal mucosal barrier function.

**Methods:**

A total of 116 children diagnosed with post-infectious cough due to lung heat and Yin deficiency were enrolled and randomly assigned to either a XBZY group or a Montelukast Sodium control group. Both groups received treatment for 10 consecutive days. Clinical outcomes were assessed using the Cough Evaluation Test, Visual Analogue Scale, Traditional Chinese Medicine (TCM) syndrome scores, and the Chinese version of the Leicester Cough Questionnaire, both before and after treatment. In addition, serum D-lactic acid levels were measured as an indicator of intestinal permeability. Fecal samples were collected to quantify the abundance of *Lactobacillus*, *Bifidobacterium*, and *Escherichia coli*, and to determine the levels of short-chain fatty acids, including acetate, propionate, and butyrate.

**Results:**

Both groups demonstrated significant improvements in clinical symptoms after treatment. Compared to the Montelukast Sodium group, children in XYZB group exhibited significantly lower Visual Analogue Scale and TCM syndrome scores, as well as higher Leicester Cough Questionnaire scores. The mean cough disappearance time was shorter, and the cough disappearance rate was significantly higher in XBZY group. Furthermore, serum D-lactic acid levels decreased significantly after treatment in XBZY group, while levels of *Lactobacillus* and Bifidobacterium increased and Escherichia coli decreased. Short-chain fatty acid concentrations, including acetate, propionate, and butyrate, were also significantly elevated in the XYZB group compared to controls.

**Conclusion:**

This exploratory study suggests that XBZY may be a promising treatment option for children with post-infectious cough characterized by lung heat and Yin deficiency. Compared with Montelukast Sodium, XBZY demonstrated superior short-term improvements in cough resolution, symptom relief, quality of life, intestinal barrier function, and gut microbiota modulation, while maintaining good safety. These findings highlight the potential role of the lung–gut axis in pediatric respiratory care and support the integration of TCM-based strategies; however, larger, blinded, and longer-term studies are needed to validate these results and assess their broader clinical applicability.

## 1. Introduction

Post-infectious cough (PIC) is one of the leading causes of chronic cough in children, particularly between the ages of 1 and 6 years.^[1]^ Defined as a persistent cough lasting more than 4 weeks after resolution of an acute upper respiratory tract infection, PIC can substantially disrupt sleep, appetite, school performance, and overall quality of life.^[2]^ Despite its clinical importance, effective treatments remain limited. The Guidelines for the Diagnosis and Treatment of Chronic Cough in Chinese Children recommend leukotriene receptor antagonists such as Montelukast and inhaled corticosteroids, but these carry only Level C recommendations, indicating that supporting evidence is weak.^[3]^ Thus, PIC represents an area of unmet clinical need where safe, effective, and evidence-based therapies are urgently required.

From a traditional Chinese medicine (TCM) perspective, the symptoms of PIC, dry or irritating cough, sometimes with scant sticky sputum, are categorized under “prolonged cough” or “chronic cough.” Many pediatric patients present with a syndrome of lung heat and Yin deficiency, often accompanied by gastrointestinal manifestations such as dry stools or constipation.^[4–7]^ This reflects the classical concept of lung–gut interaction: excess heat in the lungs can deplete Yin fluids, impair intestinal motility, and exacerbate cough. Such overlap between respiratory and digestive symptoms has been observed clinically in children with PIC.^[5,6]^

Xie Bai Zeng Ye Decoction (XBZY), a formula derived from Xiebai San and Zengye Tang, is designed to clear lung heat, nourish Yin, relieve cough, and moisten the intestines.^[8–10]^ By targeting both pulmonary and intestinal pathways, it may help break the cycle of persistent cough and gastrointestinal dysfunction. Preliminary pharmacological studies have suggested that XBZY and related formulas can modulate inflammatory signaling and improve airway responsiveness, while also exerting regulatory effects on intestinal function.^[8,9]^ These findings provide a mechanistic rationale for its application in children with PIC due to lung heat and Yin deficiency.

Based on these theoretical and experimental foundations, this study aimed to evaluate the clinical efficacy and safety of XBZY compared with Montelukast Sodium in children with PIC characterized by lung heat and Yin deficiency. The primary outcome was cough disappearance, assessed by time to cough resolution and cough resolution rate during the 10-day treatment. Secondary outcomes included cough severity scores (cough evaluation test [CET], visual analogue scale [VAS]), the Chinese version of the Leicester Cough Questionnaire (LCQ), TCM syndrome scores, and mechanistic indicators along the lung–gut axis, including serum D-lactic acid, gut microbiota composition, and fecal short-chain fatty acids.

## 2. Methods

### 2.1. Study design and ethical considerations

This study was designed as a prospective study to evaluate the comparative efficacy and safety of a modified TCM decoction versus Montelukast Sodium Tablets in the treatment of post-infectious cough in children. The sample size was estimated using G*Power 3.1.9.7. The minimum sample size was calculated to be 50, based on medium effect size of 0.43, significance level α of 0.05, power (1-β) of 0.95.^[11]^ A total of 116 participants were requested concerning ~20% rate of loss of follow-up. The study was conducted at Shenzhen Qianhai Shekou Free Trade Zone Hospital between, and was approved by the hospital’s Ethics Committee (No. 2021-KY-001-01K). Written informed consent was obtained from the parents or legal guardians of all enrolled children under the age of 18, in accordance with the principles outlined in the Declaration of Helsinki.

### 2.2. Participant recruitment and diagnostic criteria

Children aged 3 to 6 years with post-infectious cough were recruited based on a combination of Western medicine and TCM diagnostic criteria. According to the Guidelines for the Diagnosis and Treatment of Chronic Cough in Chinese Children (2013 Revision), the Western medicine diagnostic criteria required the following: a clearly documented history of recent upper or lower respiratory tract infection; a persistent cough lasting longer than 4 weeks, characterized by a dry, irritating nature or accompanied by a small quantity of viscous sputum; no significant abnormalities on chest radiography, or the presence of only mildly increased pulmonary markings; normal pulmonary ventilation function or transient airway hyperresponsiveness; a self-limiting course of illness, whereby a cough persisting longer than 8 weeks warranted the exclusion of other etiologies; and the exclusion of other causes of chronic cough, such as asthma, pertussis, bronchiectasis, or allergic rhinitis.

In parallel, children were also evaluated according to the TCM diagnostic criteria for “pediatric cough,” based on standards provided in the Standards for Diagnosis and Efficacy Evaluation of TCM Diseases by the National Administration of Traditional Chinese Medicine and the textbook Pediatrics in Traditional Chinese Medicine (10th edition). TCM diagnosis required the presence of a primary symptom of cough, typically dry and irritating, often with minimal or no phlegm. At least one secondary symptom was also required, including dry and itchy throat, sensation of heat in the palms and soles, low-grade afternoon fever, night sweats, poor appetite, disturbed sleep, or dry stools. The diagnosis was supported by tongue and pulse examination findings, such as a red tongue with minimal or geographic coating and a thin, rapid pulse.

### 2.3. Inclusion and exclusion criteria

Eligible participants met the following inclusion criteria: age between 3 and 6 years; fulfillment of both the Western medicine and TCM diagnostic criteria as described above; provision of written informed consent by a parent or legal guardian; and good compliance from both the child and their caregiver, with the willingness and ability to complete the full treatment course and follow-up evaluations.

Exclusion criteria included the following: a duration of cough exceeding 8 weeks; axillary or ear temperature >37.5°C at the time of screening; the presence of serious systemic diseases affecting organs such as the heart, liver, kidneys, or central nervous system; known allergies or hypersensitivity to any ingredients used in the study medications; and concurrent use of other treatments that could interfere with the assessment of therapeutic efficacy. Participants were also eliminated from the study if they were later determined to be misdiagnosed or if essential medical records or outcome data were incomplete. Discontinuation criteria included the development of serious adverse events, the emergence of significant complications requiring withdrawal, or voluntary withdrawal from the study by the participants’ caregivers. A participant was considered lost to follow-up (dropout) if the caregiver could not be contacted after 3 attempts through phone or in-person visit.

### 2.4. Interventions

Children in the TCM intervention group received a modified version of XBZY, comprising Sang Bai Pi (Mulberry Root Bark, 6 g), Di Gu Pi (Lycium Bark, 6 g), Xuan Shen (Scrophularia, 6 g), Di Huang (Rehmannia Root, 5 g), Mai Dong (Ophiopogon Root, 5 g), and Gan Cao (Licorice Root, 3 g). All herbs were provided in the form of authenticated decoction pieces, manufactured and quality-verified by Guangzhou Zhixin Traditional Chinese Medicine Decoction Pieces Co., Ltd., and authenticated by Huang Xiaoyu, an attending herbalist at the hospital. The decoction was prepared in the hospital’s pharmacy. Herbs were soaked in cold water for 30 minutes, followed by 2 rounds of decoction, each lasting 25 minutes. The combined filtrate was concentrated to a final volume of 2000 mL and sealed into 100 mL daily doses, stored at 4°C. Prior to administration, each dose was warmed in a water bath and taken orally, one packet twice daily for 10 consecutive days.

Children in the control group received Montelukast Sodium Chewable Tablets (4 mg/tablet), manufactured by Hangzhou MSD Pharmaceutical Co., Ltd. (National Drug Approval No. J20130053). One tablet was administered once nightly before bedtime for a duration of 10 days. Both groups were evaluated one day prior to treatment and on the final day of the treatment period.

### 2.5. Clinical outcome measures

Cough severity and its impact on daily life were assessed using 3 validated instruments: CET, VAS, and the Chinese version of LCQ. The CET followed the simplified scoring system from the Guidelines for the Diagnosis and Treatment of Cough (2021) (Table 1), while the VAS employed a 10-point horizontal scale with scores categorized as follows: 0 (no cough), 1 to 3 (mild cough), 4 to 6 (moderate cough), and 7 to 10 (severe cough). The LCQ contained 19 questions spanning physical, psychological, and social domains. Each item was rated on a 7-point Likert scale, with total scores ranging from 3 to 21; higher scores indicated better health status and lower cough-related burden. Caregivers were trained to record daily VAS and CET scores and completed the LCQ questionnaire with their child’s input at baseline and post-treatment.

**Table 1:** cough severity scores.

Questions	None	Seldom	Sometimes	Often	All of the time
How frequently did you cough during the day?	1	2	3	4	5
Have your cough disturbed your sleep?	1	2	3	4	5
Did you have intense cough?	1	2	3	4	5
Have your cough interfered with your daily life?	1	2	3	4	5
Have your cough made you feel anxious or depressive?	1	2	3	4	5

### 2.6. TCM syndrome scoring

In accordance with national TCM guidelines, the severity of 6 common symptoms, cough, sputum production, dry stools, itchy throat, night sweats, and sleep disturbances, was scored using a 4-point scale: 0 (none), 1 (mild), 2 (moderate), and 3 (severe). Scores were recorded at baseline and at the end of the 10-day treatment period.

### 2.7. Evaluation of clinical efficacy

Clinical efficacy was assessed using 2 primary outcomes: (1) cough resolution time, defined as the number of days from treatment initiation to a sustained VAS score below 1 for more than 24 hours, and (2) cough resolution rate, defined as the percentage of participants in each group meeting the resolution criterion. Treatment efficacy was further classified according to the Guidelines for Clinical Research on New Chinese Medicines. Outcomes were defined as: cured (≥95% reduction in TCM syndrome score with symptom disappearance), significantly effective (70–94% reduction), effective (30–69% reduction), and ineffective (<30% reduction). The total effective rate was calculated as the sum of cured, significantly effective, and effective cases divided by the total number of cases, multiplied by 100%.

### 2.8. Serum D-lactic acid assay

To assess changes in intestinal permeability and systemic inflammation, fasting venous blood samples were collected in the morning prior to and after the treatment course. Serum D-lactic acid levels were measured using a commercially available ELISA kit (catalog no. 2H-KMLJh310129, Camilo, China) according to the manufacturer’s protocol.

### 2.9. Gut microbiota analysis

Fresh morning stool samples (1 g) were collected from each participant before and after the treatment course. Samples were diluted with sterile physiological saline to a final dilution of 10⁻⁸ and inoculated onto selective media for Lactobacillus, Bifidobacterium, and Escherichia coli. The samples were incubated at 37°C for 48 hours. Bacterial colonies were identified using the ATB semi-automated microbiology analyzer (BioMérieux, France), and microbial abundance was expressed as the logarithmic value of colony-forming units per gram of stool (log CFU/g).

### 2.10. Short-chain fatty acid (SCFA) quantification

For SCFA analysis, 50 mg of fresh stool was collected into sterile EP tubes and stored in liquid nitrogen until analysis. From this, 25 mg of stool was homogenized in 500 μL of 0.5% phosphoric acid in water using a freeze-grinding procedure (3 minutes at 50 Hz, twice), followed by 10 minutes of ultrasonic extraction. The samples were centrifuged at 13,000 g for 15 minutes at 4°C. The resulting supernatant was mixed with 0.2 mL of n-butanol containing 2-ethylbutyric acid (10 μg/mL) as an internal standard. After 10 minutes of low-temperature ultrasonic extraction and a second centrifugation, the supernatant was analyzed using gas chromatography-mass spectrometry to quantify concentrations of acetate, butyrate, and propionate.

### 2.11. Safety evaluation

Participants’ vital signs, including temperature, respiratory rate, heart rate, blood pressure, and pulse, were monitored throughout the study. Liver and kidney function tests, as well as electrocardiograms, were performed when clinically indicated. All adverse events were documented, and their severity and potential relationship to the intervention were assessed by the clinical research team.

### 2.12. Statistical analysis

Data analysis was conducted using SPSS software version 22.0 (IBM Corp., Armonk). Children who were excluded before treatment, withdrew, or were lost to follow-up were not included in the final analysis. No data imputation was performed because the proportion of missing data was small (<10% in each group) and missingness was primarily due to voluntary withdrawal or noncompliance rather than random loss. The normality of the data was determined via the Kolmogorov–Smirnov test, and the homogeneity of variances was checked through graphical inspection of residuals. Continuous variables were expressed as mean ± standard deviation and analyzed using independent sample *t*-tests for between-group comparisons. Categorical variables were analyzed using chi-square tests. A *P*-value of <.05 was considered statistically significant.

## 3. Results

### 3.1. Participant enrollment and baseline characteristics

**Figure 1.**
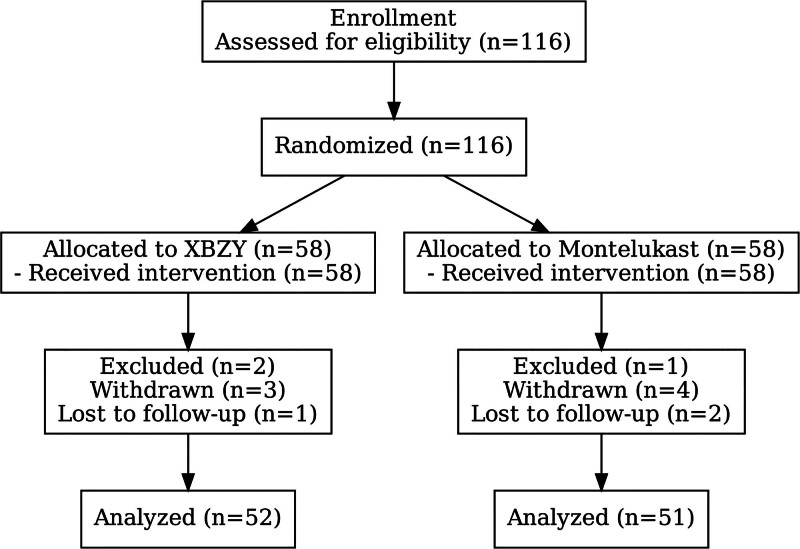
Flow diagram of the study.

Between March 2021 and March 2023, a total of 116 pediatric patients diagnosed with post-infectious cough were recruited from the Traditional Chinese Medicine outpatient department at Shenzhen Qianhai Shekou Free Trade Zone Hospital. Participants were randomized 1:1 to XBZY or Montelukast Sodium group using a computer-generated sequence with permuted blocks of variable size (4 and 6) to preserve balance. The sequence was created by an independent statistician not involved in recruitment, treatment, or outcome assessment, using a fixed random seed. Allocation was concealed with sequentially numbered, opaque, sealed envelopes. In the XBZY group, 2 children were excluded due to ineligibility, 3 discontinued treatment due to noncompliance or voluntary withdrawal, and 1 child was lost to follow-up, resulting in 52 children who completed the study. In the Montelukast Sodium group, 1 child was excluded, 4 discontinued, and 2 were lost to follow-up, yielding a final sample of 51 participants. Accordingly, the per-protocol analysis set included 52 children in the XBZY group and 51 in the Montelukast Sodium group. The study flow is presented in Figure 1.

Baseline characteristics, including age, sex distribution, and duration of illness, were comparable between the 2 groups, with no statistically significant differences observed (Table 2, *P* > .05). Similarly, there were no significant differences in baseline scores for the CET, VAS, or TCM syndrome assessment between the groups, indicating good comparability prior to intervention (Table 2).

**Table 2:** Comparison of baseline data between the 2 groups.

Group	Gender (M/F)	Age (yr)	Duration (wk)	CET score	VAS score	TCM syndrome score
XBZY	28/24	5.1 ± 1.25	6.33 ± 2.45	16.39 ± 4.23	6.29 ± 1.38	9.27 ± 3.58
Montelukast Sodium	25/26	5.3 ± 1.04	6.07 ± 1.89	15.78 ± 3.42	5.95 ± 1.75	9.38 ± 3.64

### 3.2. Improvements in cough severity, TCM syndrome scores, and quality of life

Following the 10-day treatment period, both groups demonstrated statistically significant improvements in cough severity as measured by CET and VAS scores compared to their respective baseline values (Table 3; *P* < .01). While both groups improved, the reduction in VAS score in the XBZY group was significantly greater than in the Montelukast Sodium group (Table 3; *P* < .05). However, no significant difference was found between groups in the CET scores post-treatment (Table 3; *P* > .05).

**Table 3:** Comparison of CET scores and VAS scores before and after treatment between the 2 groups.

Group	Number		CET score	VAS score
XBZY	52	before	16.39 ± 4.23	6.29 ± 1.38
		after	6.11 ± 1.09**	0.68 ± 0.12**^, △^
		95% CI (within group)	−11.48, −9.078	−5.991, −5.229
Montelukast Sodium	51	before	15.78 ± 3.42	5.95 ± 1.75
		after	6.35 ± 1.16**	1.33 ± 0.48**
		95% CI (within group)	−10.43, −8.43	−5.11, −4.13
		95% CI (inter group)	−0.6799, 0.1999	−0.7862, −0.5138

**P < .01 within-group comparison, ^△^*P* < .05 between-group comparison.

Assessment of TCM syndrome scores revealed significant improvement in both groups after treatment (Table 4; *P* < .01 or *P* < .05). The magnitude of reduction was significantly greater in the XBZY group than in the Montelukast Sodium group (Table 4; *P* < .05). Importantly, analysis of individual symptoms showed a clear divergence between groups: in the Montelukast Sodium group, dry stool, night sweats, and sleep disturbances did not improve significantly (Table 4; *P* > .05), whereas children treated with XBZY exhibited significant improvements in all 3 domains (Table 4; *P* < .05), with post-treatment scores significantly lower than those of the Montelukast Sodium group (Table 4).

**Table 4:** Comparison of TCM symptom scores before and after treatment in 2 groups.

TCM symptom	Group	Number	Before	After	95% CI (within group)
Cough	XBZY	52	2.41 ± 0.99	0.60 ± 0.45^**^	−2.11, −1.51
	Montelukast Sodium	51	2.37 ± 1.08	0.74 ± 0.69^**^	−1.98, −1.28
	95% CI (Inter group)			−0.37, 0.09	
Sputum production	XBZY	52	1.11 ± 0.41	0.23 ± 0.07*	−0.99, −0.77
	Montelukast Sodium	51	0.91 ± 0.21	0.34 ± 0.12*	−0.64, −0.50
	95% CI (Inter group)			−0.15, −0.07	
Dry stool	XBZY	52	2.23 ± 0.78	0.08 ± 0.01^**△^	−2.36, −1.94
	Montelukast Sodium	51	2.52 ± 0.74	2.34 ± 0.83	−0.49, 0.13
	95% CI (Inter group)			−2.49, −2.03	
Dry and itchy throat	XBZY	52	1.27 ± 0.61	0.14 ± 0.03^**^	−1.30, −0.96
	Montelukast Sodium	51	1.18 ± 0.41	0.41 ± 0.17*	−0.89, −0.65
	95% CI (Inter group)			−0.32, −0.22	
Sleep disturbances	XBZY	52	1.01 ± 0.39	0.15 ± 0.05^**△^	−0.97, −0.75
	Montelukast Sodium	51	1.23 ± 0.62	0.89 ± 0.11	−0.51, −0.17
	95% CI (Inter group)			−0.77, −0.71	
Night sweats	XBZY	52	1.27 ± 2.63	0.33 ± 0.42^**△^	−1.66, −0.22
	Montelukast Sodium	51	1.34 ± 0.51	1.02 ± 0.39	−0.50, −0.14
	95% CI (Inter group)			−0.85, −0.53	
Total score	XBZY	52	9.27 ± 2.63	1.33 ± 0.42^**△^	−8.66, −7.22
	Montelukast Sodium	51	9.38 ± 2.74	5.74 ± 1.77*	−4.54, −2.74
	95% CI (Inter group)			−4.91, −3.91	

* *P* < .05, ** *P* < .01 within-group comparison, ^△^*P* < .05 between-group comparison.

In terms of quality of life, both groups showed significant improvements in the physical, psychological, and social dimensions of the Chinese version of LCQ following treatment (Table 5; *P* < .01). Post-treatment LCQ scores were significantly higher in the XBZY group across all 3 dimensions compared to the Montelukast Sodium group (Table 5; *P* < .05), indicating a greater positive impact on the patients’ overall well-being.

**Table 5:** Comparison of LCQ scores before and after treatment in 2 groups.

Group	Number		Physical	Psychological	Social
XBZY	52	before	4.10 ± 0.53	3.64 ± 0.43	1.60 ± 0.28
		after	7.23 ± 0.82**^△^	6.83 ± 0.76**^△^	2.91 ± 0.35**^△^
		95% CI (within group)	2.86, 3.40	2.95, 3.43	1.19, 1.43
Montelukast Sodium	51	before	4.22 ± 0.51	3.62 ± 0.43	1.59 ± 0.22
		after	6.25 ± 0.73**	6.05 ± 0.63**	2.31 ± 0.28**
		95% CI (within group)	1.79, 2.27	2.22, 2.64	0.62, 0.82
		95% CI (inter group)	0.74, 1.46	0.44, 1.08	0.43, 0.75

** *P* < .01 within-group comparison, ^△^*P* < .05 between-group comparison.

### 3.3. Comparison of treatment efficacy

The mean time to cough resolution, defined as a sustained VAS score of <1 for over 24 hours, was significantly shorter in the XBZY group compared to the Montelukast Sodium group (Table6; *P* < .05). Additionally, the cough resolution rate in the XBZY group was 73.5%, significantly higher than the 51.0% observed in the Montelukast Sodium group (Table 6; *P* < .01).

**Table 6:** Comparison of cough disappearance time and cough disappearance rate between the 2 groups.

Group	Number	Cough disappearance time /day	cough disappearance rate /%
XBZY	52	7.77 ± 0.68^△^	73.5^△△^
Montelukast Sodium	51	9.38 ± 0.57	28.97
	95% CI (inter group)	−1.86, −1.36	

△ *P* < .05, ^△△^ *P* < .01 between-group comparison.

Evaluation of overall treatment efficacy, based on reductions in TCM syndrome scores, demonstrated that the total effective rate in the XBZY group was 92.31% (48/52), which was significantly higher than the 78.85% (41/51) observed in the Montelukast Sodium group (Table 7; *P* < .01). These findings indicate that the herbal formulation was not only faster-acting but also more effective in reducing clinical symptoms associated with post-infectious cough.

**Table 7:** Comparison of effective rate between the 2 groups.

Group	Number	Cured	Significantly Effective	Effective	Ineffective	Effective rate/%
XBZY	52	12	27	9	4	92.31%^△△^
Montelukast Sodium	51	3	15	23	10	78.85%

△△ *P* < .01 between-group comparison.

### 3.4. Serum D-lactic acid and gut microbiota composition

At baseline, no significant differences in serum D-lactic acid levels were observed between the 2 groups. After treatment, serum D-lactic acid levels remained unchanged in the Montelukast Sodium group (Table 8; *P* > .05). In contrast, the XBZY group exhibited a significant reduction in serum D-lactic acid levels post-treatment (Table 8; *P* < .01), with levels significantly lower than those observed in the Montelukast Sodium group at the same time point (Table 8; *P* < .01). These findings suggest an improvement in intestinal barrier function following herbal treatment.

**Table 8:** Comparison of serum D-lactic acid levels before and after treatment between the 2 groups (μmol/L).

Group	Number	Before	After	95% CI (within group)
XBZY	52	12.17 ± 2.73	8.98 ± 1.45**^△△^	−4.03, −2.35
Montelukast Sodium	51	13.33 ± 3.12	12.11 ± 2.31	−2.29, −0.16
	95% CI (inter group)		−3.33, −0.61	

** *P* < .01 within-group comparison, ^△△^*P* < .01 between-group comparison.

Microbiological analysis of stool samples revealed no significant differences in the composition of gut microbiota between the groups at baseline. However, after treatment, the XBZY group demonstrated a significant increase in beneficial bacteria, including Lactobacillus and Bifidobacterium species (Table 9; *P* < .05), and a significant reduction in Escherichia coli levels (Table 9; *P* < .05). These changes were significantly different from those observed in the Montelukast Sodium group (Table 8; *P* < .05). While the Montelukast Sodium group showed an upward trend in beneficial bacteria and a downward trend in *E. coli* levels, these changes were not statistically significant compared to baseline values (Table 9).

**Table 9:** Comparison of gut microbiota composition before and after treatment between the 2 groups (lg CFU/g).

Group	Number		Lactobacillus	Bifidobacterium	*Escherichia coli*
XBZY	52	Before	7.70 ± 1.88	7.65 ± 2.04	8.95 ± 2.06
		After	9.37 ± 1.97*^△^	9.86 ± 1.31*^△^	8.01 ± 1.24*^△^
		95% CI (within group)	1.31, 2.03	1.75, 2.68	−1.43, −0.45
Montelukast Sodium	51	Before	7.68 ± 1.52	7.67 ± 1.72	8.93 ± 2.11
		After	8.36 ± 1.83	8.41 ± 1.91	8.65 ± 1.36
		95% CI (within group)	0.38, 1.00	0.49, 1.00	−0.59, −0.01
		95% CI (inter group)	0.50, 1.40	0.79, 1.93	−1.26, −0.23

* *P* < .05 within-group comparison, ^△^*P* < .05 between-group comparison.

### 3.5. SCFA levels

Analysis of SCFA profiles in stool samples revealed no significant differences between groups at baseline. Following treatment, children in the XBZY group exhibited significant increases in the levels of acetate, butyrate, and propionate compared to baseline (Table 10; *P* < .05). These increases were also significantly greater than those observed in the Montelukast Sodium group (Table 10; *P* < .05). In contrast, no significant changes in SCFA levels were detected in the Montelukast Sodium group following treatment (Table 10). The elevated SCFA levels in the herbal treatment group suggest improved gut microbial metabolism and intestinal health.

**Table 10:** Comparison of short-chain fatty acids before and after treatment in 2 groups (μg/g).

Group	Number		Acetate	Butyrate	Propionate
XBZY	52	Before	5.89 ± 1.23	2.69 ± 0.61	6.50 ± 1.61
		After	7.72 ± 1.73*^△^	4.03 ± 0.78*^△^	7.74 ± 1.98*^△^
		95% CI (within group)	1.40, 2.26	1.13, 1.54	0.66, 1.82
Montelukast Sodium	51	Before	5.95 ± 1.12	2.45 ± 0.53	6.63 ± 1.58
		After	6.49 ± 1.18	2.33 ± 0.56	6.71 ± 1.47
		95% CI (within group)	0.29, 0.79	−0.18, −0.06	−0.06, 0.22
		95% CI (inter group)	0.65, 1.49	1.25, 1.65	0.24, 1.11

* *P* < .05 within-group comparison, ^△^*P* < .05 between-group comparison.

### 3.6. Safety evaluation

Throughout the study period, no serious adverse events, drug-related adverse reactions, or other unexpected safety concerns were reported in either group. Routine monitoring of vital signs and, where indicated, laboratory assessments including liver and renal function and electrocardiograms, revealed no abnormalities attributable to either treatment. Both interventions were well tolerated by the participants, and compliance was generally good.

## 4. Discussion

This study demonstrates that the application of XBZY, based on the TCM principle of combined lung and intestinal treatment, provides therapeutic benefits for children with PIC presenting with lung heat and Yin deficiency. The treatment accelerated cough resolution, improved gastrointestinal symptoms such as constipation and night sweats, and enhanced overall quality of life. Compared to Montelukast Sodium, XBZY showed advantages on several outcomes (VAS, LCQ, and cough resolution time), whereas CET did not differ significantly between groups. These findings suggest that XBZY may complement or provide an alternative to conventional therapy, though superiority is not supported across all endpoints.

XBZY, a modified formula combining Xiebai San and Zengye Tang, was designed to target both pulmonary and intestinal manifestations. Classical herbal components such as Sang Bai Pi and Xuan Shen clear lung heat, enrich Yin, and moisten the intestines, while adjunct herbs restore fluid balance and harmonize Qi.^[12]^ Prior studies have demonstrated immunomodulatory effects of Xiebai San through PI3K–AKT pathway regulation, while Zengye Tang has been reported to modulate gut microbiota.^[13–16]^ Our clinical results are consistent with these mechanistic observations, with XBZY producing significant improvements in cough resolution, syndrome-related symptoms, and quality-of-life scores.

From a biomedical perspective, the concept of the “lung–gut axis” offers a modern framework for understanding these outcomes. This axis involves complex communication between the respiratory and gastrointestinal mucosa via the common mucosal immune system, which includes lymphoid tissues from the lungs, gut, and other mucosal surfaces.^[17,18]^ Upon activation by pulmonary pathogens, immune cells migrate systemically, with some homing to the gut, where they may trigger inflammation and disrupt barrier function.^[19,20]^ One marker of intestinal barrier disruption is D-lactic acid, a metabolite produced by intestinal bacteria. When the intestinal mucosa is damaged, D-lactic acid translocates into the bloodstream, making it a sensitive biomarker of intestinal permeability.^[21]^ In our study, serum D-lactic acid levels significantly decreased in the TCM group following treatment, indicating improved intestinal barrier integrity, an effect not observed in the Montelukast Sodium group.

The restoration of intestinal health was further supported by gut microbiota analysis. The TCM group showed a significant post-treatment increase in beneficial microbes, including Lactobacillus and Bifidobacterium, and a concurrent reduction in *Escherichia coli*, a marker of dysbiosis.^[22]^ Lactobacillus and Bifidobacterium have well-established roles in reducing inflammation and modulating allergic responses through mechanisms such as mitogen-activated protein kinase pathway inhibition and suppression of pro-inflammatory chemokines.^[23]^ In contrast, Escherichia coli can stimulate Th1 cytokine production, disturb the Th1/Th2 balance, and contribute to immune dysfunction and disease progression.^[24]^ The observed microbial changes thus signify a return to a more balanced and health-promoting intestinal ecosystem.

Furthermore, the study assessed SCFAs, the primary metabolites of gut bacteria involved in mucosal health and immune regulation. SCFAs such as acetate, propionate, and butyrate support epithelial integrity, modulate immune cell activity, and activate G protein-coupled receptors that suppress inflammation. Butyrate, in particular, upregulates G protein–coupled receptor 41 (GPR41) and G protein–coupled receptor 43 (GPR43), reduces pro-inflammatory T-cell proliferation, and promotes tissue repair via neutrophil activation. Acetate and propionate enhance the number and function of regulatory T cells, thereby attenuating airway inflammation.^[25–27]^ In our study, children treated with XBZY exhibited significantly elevated levels of all 3 SCFAs post-treatment, indicating enhanced gut microbial metabolism and further supporting the observed immunomodulatory effects.

Several methodological considerations qualify these conclusions. The lack of blinding represents a major limitation, particularly for subjective endpoints such as VAS and LCQ, and may have introduced expectation or observer bias. The short follow-up window precludes assessment of durability, relapse, or longer-term quality-of-life effects. Differences across measurement instruments also matter: although XBZY improved VAS and LCQ, the absence of a between-group effect on CET suggests that this categorical tool may be less sensitive to short-term change or that the study was underpowered to detect modest differences on that endpoint. In addition, we did not perform covariate-adjusted regression analyses. This decision was based on the randomized design and good baseline balance across key characteristics (age, sex, illness duration, and baseline CET/VAS/LCQ/TCM scores), which reduces the likelihood of meaningful confounding. Given the exploratory nature of the study and the modest sample size, fitting multivariable models with several covariates risked overfitting and unstable estimates without a clear validity gain. Nevertheless, we acknowledge the possibility of residual confounding from unmeasured factors; future multicenter studies with larger samples should be prospectively powered for prespecified, covariate-adjusted and sensitivity analyses to probe robustness. Finally, generalizability is constrained by the inclusion of children with a specific TCM syndrome pattern, lung heat and Yin deficiency, limiting direct application to broader PIC populations or other syndrome types.

## 5. Conclusions

In conclusion, this exploratory study suggests that XBZY may be a promising treatment option for post-infectious cough in children with lung heat and Yin deficiency. Compared with Montelukast Sodium, XBZY showed superior short-term improvements in symptom relief, quality of life, intestinal barrier function, and gut microbiota modulation, while maintaining a favorable safety profile. These findings support the potential role of the lung–gut axis as a mechanistic pathway and highlight the value of integrating TCM-based approaches into pediatric respiratory care. However, given the short follow-up, absence of blinding, and syndrome-specific inclusion, the evidence should be interpreted cautiously. Multicenter, larger, blinded, and longer-term trials are essential to confirm these results, clarify causality, and determine the broader clinical applicability of this therapeutic strategy.

## Author contributions

**Conceptualization:** Yi Ding.

**Data curation:** Jing Luo, Dan Zhang, Yi Ding.

**Formal analysis:** Jing Luo, Dan Zhang, Yi Ding.

**Funding acquisition:** Yi Ding.

**Investigation:** Jing Luo, Dan Zhang.

**Project administration:** Guoping Zhan.

**Resources:** Guoping Zhan.

**Software:** Guoping Zhan.

**Writing – original draft:** Jing Luo, Dan Zhang, Guoping Zhan, Yi Ding.

**Writing – review & editing:** Jing Luo, Dan Zhang, Guoping Zhan, Yi Ding.

## References

[1] Enmei L Quan L. Clinical practice guidelines for the diagnosis and management of children with cough in China (version 2021). Zhonghua Er Ke Za Zhi. 2021;59:720–9. 34645211 10.3760/cma.j.cn112140-20210513-00423

[2] Waring G Kirk S Fallon D. The impact of chronic non-specific cough on children and their families: a narrative literature review. J Child Health Care. 2020;24:143–60. 30606033 10.1177/1367493518814925

[3] Quan L. Guideline for diagnosis and treatment of chronic cough in Chinese children. Zhonghua Er Ke Za Zhi3. 2014;52:184–8. 24824387

[4] Yuan M Chen H Rui W. The research advance on the theory of Chinese medicine-exterior-interior correlation between the Lung and Large Intestine in the treatment of chronic cough. J Holistic Integr Pharm. 2024;5:195–204.

[5] Stricker S Hain T Chao C-M Rudloff S. Respiratory and intestinal microbiota in pediatric lung diseases-current evidence of the gut-lung axis. Int J Mol Sci. 2022;23:6791. 35743234 10.3390/ijms23126791 PMC9224356

[6] Cheepsattayakorn A Cheepsattayakorn R. Parasitic pneumonia and lung involvement. Biomed Res Int. 2014;2014:874021. 24995332 10.1155/2014/874021 PMC4068046

[7] Sun Y Zhao Y Xue SA Chen J. The theory development of traditional Chinese medicine constitution: a review. J Tradit Chin Med Sci. 2018;5:16–28.

[8] Luo J Deng Y Ding Y Tang C Wang M. Xiebai Zengye decoction improves respiratory function and attenuates inflammation in juvenile rats with postinfection cough via regulating ERK signaling pathway. Cell Biochem Funct. 2023;41:857–67. 37606071 10.1002/cbf.3835

[9] Luo J Deng Y Ding Y. Investigation into actions of Xiebai and Zengye decoction on cough sensitivity, airway inflammation and gut microbiota in the rat model of post-infectious cough. Heliyon. 2023;9:e22782. 38094068 10.1016/j.heliyon.2023.e22782 PMC10716551

[10] Zhou B-W Liu H-M Jia X-H. The role and mechanisms of traditional Chinese medicine for airway inflammation and remodeling in asthma: overview and progress. Front Pharmacol. 2022;13:917256. 35910345 10.3389/fphar.2022.917256 PMC9335520

[11] Faul F Erdfelder E Buchner A Lang A-G. Statistical power analyses using G*Power 3.1: tests for correlation and regression analyses. Behav Res Methods. 2009;41:1149–60. 19897823 10.3758/BRM.41.4.1149

[12] Zhao A Guo C Wang L. Xiebai San alleviates acute lung injury by inhibiting the phosphorylation of the ERK/Stat3 pathway and regulating multiple metabolisms. Phytomedicine. 2024;128:155397. 38547623 10.1016/j.phymed.2024.155397

[13] Guo Z Yin H Wu T. Study on the mechanism of Cortex Lycii on lung cancer based on network pharmacology combined with experimental validation. J Ethnopharmacol. 2022;293:115280. 35405252 10.1016/j.jep.2022.115280

[14] Liu Y Kong H Cai H Chen G Chen H Ruan W. Progression of the PI3K/Akt signaling pathway in chronic obstructive pulmonary disease. Front Pharmacol. 2023;14:1238782. 37799975 10.3389/fphar.2023.1238782 PMC10548138

[15] Shi LL Li Y Wang Y Feng Y. MDG-1, an Ophiopogon polysaccharide, regulate gut microbiota in high-fat diet-induced obese C57BL/6 mice. Int J Biol Macromol. 2015;81:576–83. 26321425 10.1016/j.ijbiomac.2015.08.057

[16] Jiang JG Luo Q Li S-S. Cinnamic acid regulates the intestinal microbiome and short-chain fatty acids to treat slow transit constipation. World J Gastrointest Pharmacol Ther. 2023;14:4–21. 36911598 10.4292/wjgpt.v14.i2.4 PMC9993904

[17] Dang AT Marsland BJ. Microbes, metabolites, and the gut-lung axis. Mucosal Immunol. 2019;12:843–50. 30976087 10.1038/s41385-019-0160-6

[18] Zhu W Wu Y Liu H Jiang C Huo L. Gut-lung axis: microbial crosstalk in pediatric respiratory tract infections. Front Immunol. 2021;12:741233. 34867963 10.3389/fimmu.2021.741233 PMC8637285

[19] Zhang Y Wan Y Xin X. Signals from intestinal microbiota mediate the crosstalk between the lung-gut axis in an influenza infection mouse model. Front Immunol. 2024;15:1435180. 39114658 10.3389/fimmu.2024.1435180 PMC11304505

[20] Sun M Lu F Yu D Wang Y Chen P Liu S. Respiratory diseases and gut microbiota: relevance, pathogenesis, and treatment. Front Microbiol. 2024;15:1358597. 39081882 10.3389/fmicb.2024.1358597 PMC11286581

[21] Cai Y Gong D Xiang T Zhang X Pan J. Markers of intestinal barrier damage in patients with chronic insomnia disorder. Front Psychiatry. 2024;15:1373462. 38606411 10.3389/fpsyt.2024.1373462 PMC11007705

[22] Średnicka P Roszko ML Popowski D. Effect of in vitro cultivation on human gut microbiota composition using 16S rDNA amplicon sequencing and metabolomics approach. Sci Rep. 2023;13:3026. 36810418 10.1038/s41598-023-29637-2 PMC9945476

[23] Li SC Lin H-P Chang J-S Shih C-K. Combination of *Lactobacillus acidophilus* and *Bifidobacterium animalis* subsp. lactis shows a stronger anti-inflammatory effect than individual strains in HT-29 cells. Nutrients. 2019;11:2718. 31035617 10.3390/nu11050969 PMC6566532

[24] Chen L Ruan G Cheng Y Yi A Chen D Wei Y. The role of Th17 cells in inflammatory bowel disease and the research progress. Front Immunol. 2022;13:1055914. 36700221 10.3389/fimmu.2022.1055914 PMC9870314

[25] Du Y He C An Y. The role of short chain fatty acids in inflammation and body health. Int J Mol Sci. 2024;25:7379. 39000498 10.3390/ijms25137379 PMC11242198

[26] Yip W Hughes MR Li Y. Butyrate shapes immune cell fate and function in allergic asthma. Front Immunol. 2021;12:628453. 33659009 10.3389/fimmu.2021.628453 PMC7917140

[27] Theiler A Bärnthaler T Platzer W. Butyrate ameliorates allergic airway inflammation by limiting eosinophil trafficking and survival. J Allergy Clin Immunol. 2019;144:764–76. 31082458 10.1016/j.jaci.2019.05.002

